# Link Workers in Social Prescribing for Young People Work: A Case Study From Sheffield Futures

**DOI:** 10.5334/ijic.7551

**Published:** 2024-02-07

**Authors:** Isabel Farina, Marcello Bertotti, Cristina Masella, Daniela Sangiorgi

**Affiliations:** 1 Department of Management Engineering, Politecnico of Milan, Italy; 2 Center for Connected Care, University of East London, UK; 3 Department of Design, Politecnico of Milan, Italy

**Keywords:** Social Prescribing, Link Workers, Children and Young people

## Abstract

**Introduction:**

Social Prescribing has an established recognition regarding the benefits provided to the health-related social needs of adults, but little is known about how the intervention addresses young people’s needs. There is optimism regarding the central role of two core mechanisms that allows social prescribing to be effective, such as the empathetic role of Link Workers and the connection with community resources.

This paper aims to describe the role played by Link Workers working a Social Prescribing intervention targeting young people.

**Description:**

This paper adopts a case study methodology to describe the role of Link Workers addressing young people’s needs and implementing Social Prescribing scheme in Sheffield (UK). Data were collected through semi-structured interviews with four of the seven link workers of one organisation based in Sheffield. Data were analysed through an inductive approach for emerging themes.

**Discussion:**

We provided a description of the profiles and background of Link Workers and described the three models of referral pathways into the intervention. The paper also shows how Link Workers identify young people’s needs and how they connect with the community.

**Conclusion:**

Based on the insights and the internationally accepted definition of Social Prescribing, we provide a visual representation of the Social Prescribing model and discuss challenges. The paper highlights lessons learned and future directions regarding the role of Link Workers from the case study.

## Introduction

### Background

Young people’s wellbeing and mental health have become an increasing challenge for healthcare systems across the world [1]. It is estimated that more than 13% of adolescents live with a mental disorder [2] and approximately 75% of those conditions manifest by the age of 24 [3]. Multiple factors affect mental health during adolescence such as media influence, pressure to conform (which create a disparity between reality and perceptions), gender norms, but also socioeconomic determinants (living conditions, lack of access to support services, poverty, discrimination) and exposure to home-based violence, quality of home life and relationships with peers, school pressure [4]. The systems that should support them are facing a challenge in promoting help seeking behaviour and more youth-friendly services [5].

One intervention that is gaining popularity at a global level [6] is Social Prescribing defined as “*means for trusted individuals in clinical and community settings to identify that a person has non-medical, health-related social needs and to subsequently connect them to non-clinical supports and services within the community by co-producing a social prescription – a non-medical prescription, to improve health and wellbeing and to strengthen community connections”* [7]. More recently it has been adopted in the UK for a younger target. In 2019 the English National Healthcare system (NHS) launched the Long Term Plan, a public sector commitment to make Social Prescribing available to 900,000 people of all ages by 2023/24. In order to deliver this commitment, to date, the NHS has recruited over 1,000 link workers to support primary care referred patients across England. Despite the Long Term Plan commitment to deliver Social Prescribing for all ages (including young people), in practice, much attention from policy and research has been paid to adult Social Prescribing rather than for Children and Young People (CYP). Recently, this has begun to change with growing interest towards younger people. For instance, the Department of Health and Social Care has funded the implementation and evaluation of a Social Prescribing for CYP pilot study in three English sites (Sheffield, Luton and Brighton & Hove).

### Literature review

As described in the second paragraph above, the definition of Social Prescribing does not specifically mention Link Workers. However, authors explain the role of ‘connectors’ as non-clinical professionals that co-produce with the person an action plan and provide support to access relevant community resources. Link Workers is one of the names given to these connectors, which in some cases might also be the same as the referrer. Questions regarding the role of Link Workers were also raised in a recent review [8] whose aim was to explore the evidence base around SP to improve the mental health and wellbeing of CYP. As no studies or grey literature met inclusion criteria, no conclusions could be drawn but authors provided interesting explanations regarding the results. One finding was related to the focus of the review to the Link Workers Social Prescribing model that may neglect other methods of referral and connection leaving the question open if the Link Worker model is the preferable one for young people.

In a recent comment piece published in The Lancet [9] the authors suggest that there are good reasons to believe that two core mechanisms of adult SP may apply also for young people SP. These two mechanisms are the empathetic relationship between Link Workers and the individual. The second mechanism is the connection between individuals and community support services. Authors are optimistic about the potential value that SP can bring to children and young people with poor mental health.

The broader literature about Link Workers shows that their empathetic role is central to the achievement of wellbeing and successful linking to relevant services. This empathy is achieved by using realistic, progressive and personalised goals, social support, power balance between Link Workers and the young person, behaviour change techniques to reach self-confidence and have coping strategies [10]. Major focus is also placed on the relationship with primary care providers to ensure their buy-in, and the importance of a feedback loop between them and Link Workers. One of the implications of the feedback loop is also to increase the visibility of community resources to the primary care sector [11].

A two-year pilot evaluation study [12] concluded that Link Workers play a crucial role in the success of Social Prescribing. The same study also argued that accessibility to the service is related to Link Workers flexibility in providing support to their clients depending on their needs and aspirations, which may include service outreach. They also provide a high level of support by accompanying young people who lack the confidence to access further support and activities. Authors also present some relevant differences between Link Workers working with young people and those working with adults. Young people have a whole ecosystem of care around them, which means that Link Workers need to coordinate support provision with families and other agencies involved (i.e., schools, social workers).

This also means that they must balance confidentiality and expectations to build a trusting relationship. They often must advocate for young people and encourage them to express their needs. Link Workers have a complex role that includes managing caseload but also identifying and update their knowledge on the services and activities available in the community alongside maintaining their visibility to the different referring organisations and services.

In a recent evaluation of a Social Prescribing intervention in Leeds [13], authors focused specifically on the health and social outcomes of 16–25 years old. This evaluation investigated the role of the Link Worker and highlighting its effectiveness in reducing loneliness and social isolation, increasing behavioural activation and improving self-esteem.

Yet, the evidence regarding the role of Link Workers in the development of Social Prescribing for young people is still extremely limited. The UK government is investing in developing Social Prescribing through the role of Link Workers and there is a growing attention as a positive intervention for young people. For these reasons this paper aims to describe the role of the Link Worker working in a Social Prescribing intervention targeting young people. By describing their role the paper focuses on the empathetic relationship they built with young people, the pathways to the intervention and the connection to community support services.

This paper aims to describe the role played by Link Workers working a Social Prescribing intervention targeting young people.

### Problem statement

Social prescribing is an important example of integrated care which involves a specific type of workforce (i.e., Link Workers) who are engaged in providing health and social support across different parts of the healthcare ecosystem from primary care to the voluntary sector. Although research on Social Prescribing for adults has developed considerably, there is only very limited knowledge of the role of Link Workers in social prescribing for children and young people. This paper adopts a single case study methodology to report Link Workers’ perspectives on working with young people and on the general understanding of what Social Prescribing is by examining different pathways available to young people.

## Ethical approval

This research project did not include the collection, processing or analysis of personal or sensitive data of an interested party. Fare clic qui per immettere testo.The Politecnico of Milan ethical committee has not required ethical clearance for this study as no sensitive data has been collected and the research is part of the fieldwork for the doctoral thesis of the main author. Nevertheless, participants were asked to agree to participate in the interviews on a voluntary basis and written informed consent has been obtained for the publication of this case study only at an aggregated and/or anonymous level according to privacy policy issued under art. 13 of EU Regulation 2016/679 of 27 April 2016.

## Methodology

This paper adopts a case study methodology [14] to describe and explore a contextual Social Prescribing scheme for young people (13–25 years old) in Sheffield (UK) and delivered by an organisation called Sheffield Futures. It adopts Link Workers’ perspective from a single SP scheme to describe their role in the intervention, how Social Prescribing has to be adapted for the younger population and which elements could help to enhance its impact on young people’s wellbeing.

Single Case study methodology is particularly useful to describe and explain the mechanisms of services and organisations, and relevant actors’ role [15]. Information arising from online resources (such as Sheffield Futures website, grey literature of previous reports of Street Games) has been previously collected to understand the context. Data were collected through semi-structured interviews, lasting between 1 hour and 45 minutes and 2 hours each with four of the seven link workers of the organisation, each one delivering the Social Prescribing intervention in different settings, showing the variety of ways to engage with young people.

All interviews’ data were recorded, transcribed and analysed using an inductive coding approach. Open codes were processed in emergent themes [16]. We followed the Social Prescribing definition as an underlined narrative (referral path, co-production with young people, community connection) to understand each phase of the intervention.

### Unit of analysis: Sheffield Futures

Sheffield Futures is a charity with more than 25 years of experience in providing mental health, emotional support and sexual health advice to young people.

In the city of Sheffield, the charity is known for its drop-in one-stop-shop called Door43, defined as a YIACS (Youth Information, Advice and Counselling Service) which is accessible for young people aged 13–25 years old every day of the week. At Door43 there is a Wellbeing Cafè where young people are encouraged and supported in positive wellbeing through social and creative activities. It also provides a sexual health drop-in and clinic. The one-stop-shop is designed to welcome and engage with young people who struggle with mental health, emotional wellbeing, sexual health, housing, substance misuse, money advice and benefits, education and employability, bullying and difficulties at home. But at the same time, it is meant as a youth-friendly and safe space where young people can be listened to.

**Figure 1:**
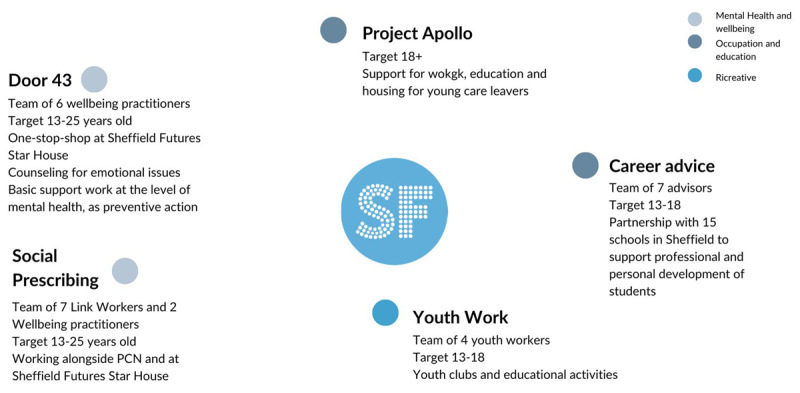
Visual description of services provided by Sheffield Futures.

Besides Door43, Sheffield Futures provides five key services as described in Figure 1: Project Apollo which supports young care leavers, Career advice working along with schools, Youth Work which develops youths’ clubs and Social Prescribing.

The way Social Prescribing is presented in Sheffield Futures’ website resonates with the general definition of SP explained above: “*Our social prescribing team works with young people aged 13–25 in Sheffield to help them with challenges such as low mood, loneliness and anxiety. Simply put, social prescribing finds non-medical ways of helping you feel better about yourself and your life. Rather than writing a prescription for a medication, a Social Prescribing Link Worker from Sheffield Futures can signpost you to a source of non-medical support in the area where you live, such as a community group or class. Taking part in sport, cycling, walking, creative arts, volunteering and dancing are all examples of activities you might be ‘prescribed’. We work with you to find out about the things you enjoy, your values and the things that matter most to you, and what’s going on in your local area*.” [15]

**Figure 2:**
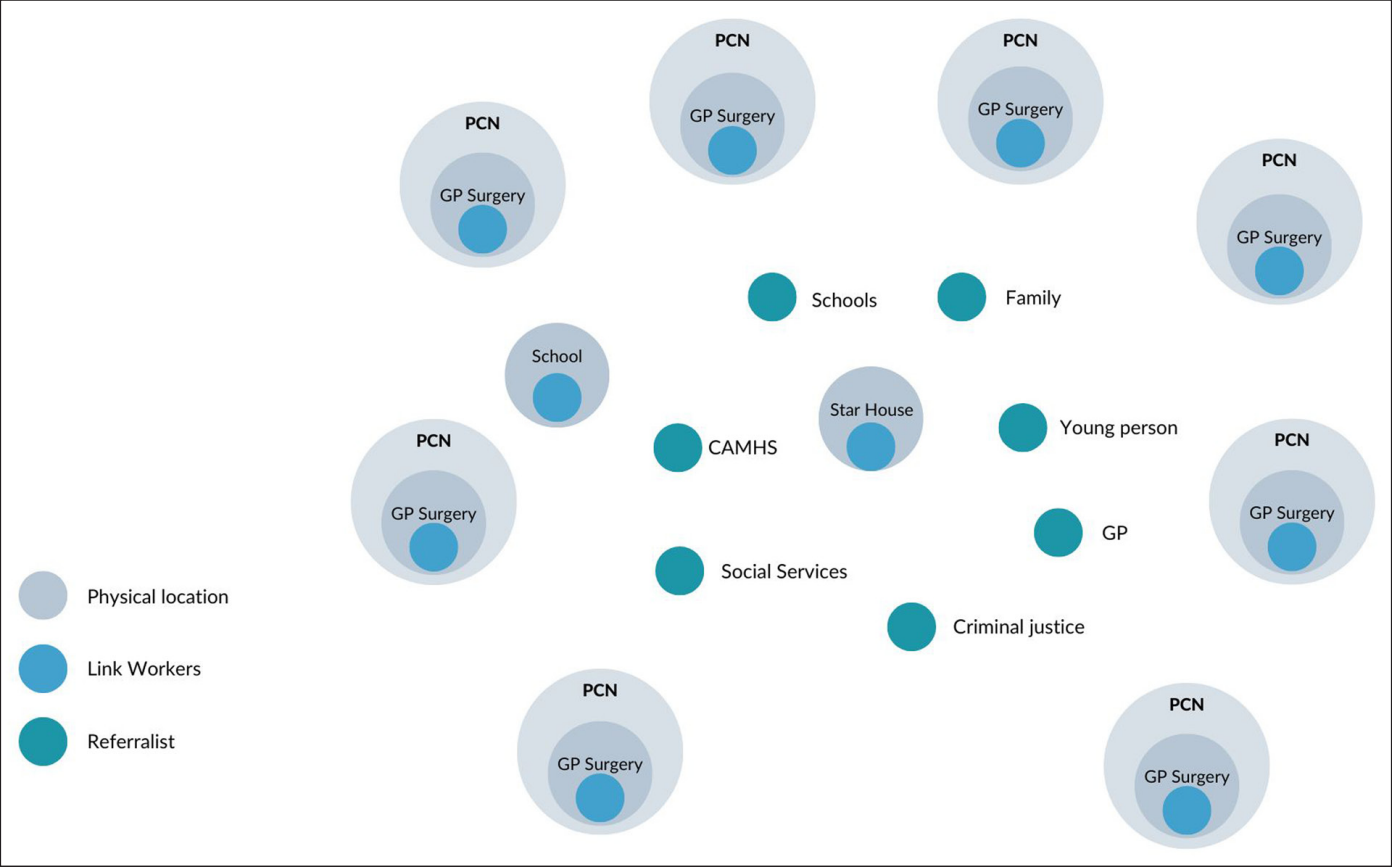
Visual representation of the model of link workers location.

At the time of the research there were seven Link Workers hired by Sheffield Futures whose role was funded by different Primary Care Networks (PCN). PCN are networks across primary care providers established by the NHS Long Term Plan in 2019 in order to share resources across General Practitioners (GPs) and develop an Integrated Care System. This means that Link Workers role depends on the funding available from PCN and that they cover the area related to their PCN, typically between 30,000 to 50,000 people. Sheffield Futures delivers Social Prescribing in eight of the fifteen Primary Care Networks in the municipality and recently also in one school. LWs are typically located in General Practitioners (GPs) surgeries. As the Social Prescribing service does not cover all PCN in Sheffield, one LW works at a central location at Sheffield Futures Star house to receive referrals across the city, especially from the more deprived areas (see Figure 2).

## Results

### Link Workers’ profiles

Four (out of seven) Link Workers from Sheffield Futures participated in the study, each representing the four main referral pathways in the organisation.

They have all been educated at degree level, having completed different types of university degrees including sport nutrition, social work or psychology. They have also volunteered in organisations delivering community and social services and have all previously worked in the healthcare and social sector. One of the respondents had already been working as Link Worker for adults in a previous job, the others had previous experience as youth worker or social worker in charities that delivered homelessness support, education, alcohol or smoke reduction, human trafficking, or domestic violence. All respondents had some work experience in their role as Link Workers in Sheffield Futures ranging from 10 months to two years.

The reasons why they decided to become Link Workers is based on a shared vision about the gaps of existing services for young people and the need to provide more rapid, personalised approaches to mental health. Those who have been working with adults were acutely aware of the need to intervene earlier, during adolescence. And those who worked with young people wanted to have a different approach.

*“I thought to myself, I’m working with adults, and I saw that a lot of the issues people live with were because of what they’d been through while they were younger.”*

*LW 1*

When applying for the role, all Link Workers received a basic two days training from the NHS which provided them with basic information about the role and how a typical session with a client looks like. The training was not focused specifically on working with young people, but it was for the support of all age groups, including adults.

Some of Link Workers integrated the initial training with more specialised training in counselling and coaching. All of them reported that the skills acquired by their previous education and work experience had been crucial to their new role.

*“I can take referrals from anywhere, including social services and youth workers, which are sort of early prevention workers for Children’s Services. Alcohol and drugs workers…and that’s all experience that I’ve gained on my social work degree, I worked with a lot of those services. And so that really helps in my role, because I can take referrals from anywhere.”*

*LW 4*

Inside the organisation, Link Workers receive regular support. Once a month the entire team meets for updates on the various projects and implementation of the service. The organisation has developed a peer support system every fifteen days through the so-called “reflective space” in which Link Workers meet without supervision, discuss their cases to share success and challenges and receive advice from colleagues. Lastly, every fifteen days the whole team meets with a primary mental health practitioner. The sessions have a focus on the mental health issues presented by young people and they are a space for technical exchange and wellbeing support for the Link Workers.

### Young people’ needs and risk assessment

**Figure 3:**
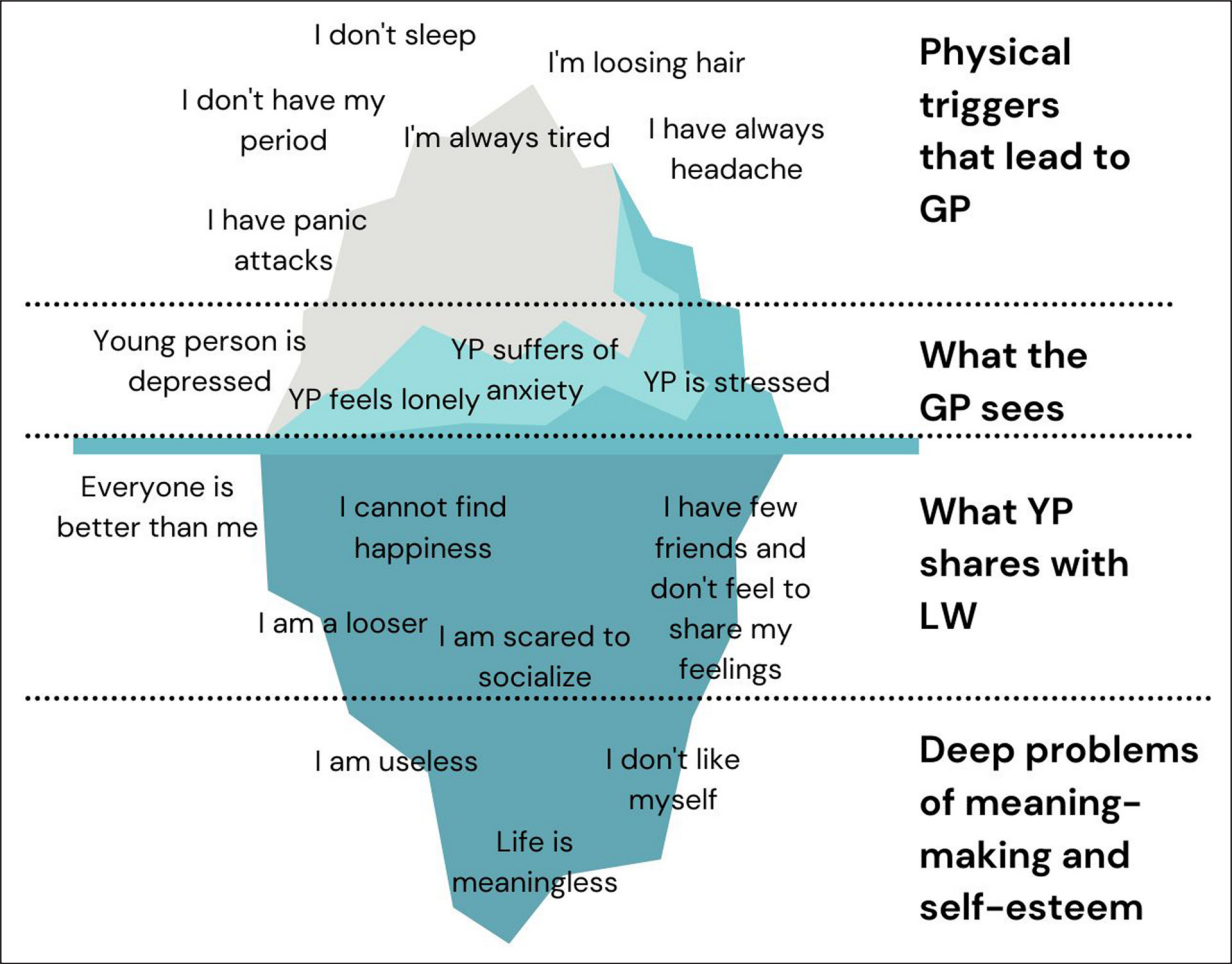
Visual synthesis from authors of the issues experienced by young people entering the Social Prescribing service from Link Workers point of view.

Each Link Worker covers a different area of the city encompassing the University, healthier neighbourhoods, and the most deprived areas. In most cases, they described common characteristics of their clients including high pressure, depression, loneliness and anxiety (Figure 3).

*“There seem to be differences all over the area, but common, it’s mental health difficulties. Seeming to be the predominant issue that we face. I think with the pandemic, obviously, children going to school from home, and then they were in the schools, and then they’re at home, and then schools and working online. That’s caused an issue, and I think it’s just them being in social isolation. So people then really lack confidence of being very anxious, being depressed because of not being able to meet other people, but then being very anxious to actually go outdoors and mix with other people.”*

*LW1*

**Figure 4:**
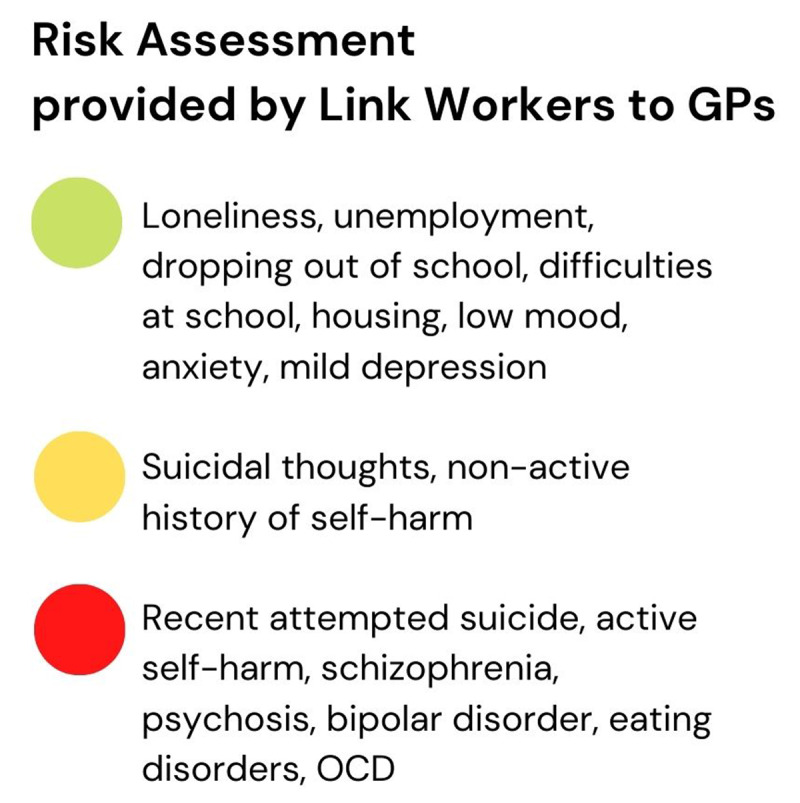
Visual synthesis of the streetlights risk assessment for referrals provided by link workers to General Practitioners.

Link workers have developed ‘streetlight’ guidelines as a risk assessment shared with GPs to ensure that they support the right level of need within the population of young people referred (Figure 4). This is an important tool as Link Workers report that the buy-in from referring organisations and the understanding of Social Prescribing are still under development.

### Social Prescribing pathways

Sheffield Futures has developed different referral pathways. These referral pathways have been explained by Link Workers during the interviews as each Link Worker interviewed reflects a differentpathway.

Referral pathway 1: The original model as set out by the Long-Term Plan, used also for adults, foresees the location of one Link Worker in a primary care setting covering all the referrals coming from the PCN. The young person is referred to the Link Worker by the GP who uses an online platform where general information about the young person and the inclusion criteria are shared. The Link Worker assesses the referral autonomously, contacts the young person, scheduling a first appointment. This type of model is based on the idea that primary care practitioners are the first identifiers of people’s social needs which resonate with a more traditional definition of Social Prescribing. However, this model may not work as effectively for young people for two reasons: evidence shows that many young people might not seek help through GPs and, in some PCN areas, a Link Worker might not be available as the service is only available in eight of fifteen PCNs in Sheffield. For these reasons, two additional models of referral have been funded by Primary Care Networks and Sheffield City Council in order to reach those young people who do not access primary care.

Referral pathway 2: The second model of referral foresees the presence of the Link Worker at Star House, Sheffield Futures’ hub, rather than at GP practice. This model of referral allows other identifiers to reach Social Prescribing and is because Sheffield Futures is a well-established organisation that provides low levels of mental health support for young people. Identifiers might not know Social Prescribing, but they recognize the organisation’s experience in providing support. The Link Worker in this case is not limited by GP practice referral: young people can self-refer or be referred by other stakeholders such as social workers, schools, and families allowing a broader accessibility for young people, especially those who do not access primary care. This model also allows to reach those who do not regularly visit GPs practices for their mental health needs and those who do not know Social Prescribing and the type of support they might need. In this case individuals might just enter Door43 or send a self-referral through the online system. The coordinators of the different services at Sheffield Futures can then identify the most appropriate service for the young person, including Social Prescribing.

*“You’re in a position where the doctors are funding a role for patients that are not attached to their surgery, but they’re doing it because they believe that the wider positive outcome will be of benefit, you know, better, happier young people in general is a benefit to all. I think that’s kind of what they’re thinking.”*

*LW 4*

This referral model aims to make Social Prescribing more flexible in terms of accessibility, considering that many young people do not access the primary care system but instead use other points of access including for instance schools and self-referral through Door43.

Referral pathway 3: In the last model, which at the time of the interviews was an experiment, one LW is in one school in Sheffield and receives students directly or through the support of teachers. This model has recently been adopted as an intervention that aims to reach and engage students at an earlier stage. The school engaged in this model was found to be the one whose students were mostly referred to LWs by the local PCN. Instead of having students in need moving from school to general practice to Social Prescribing, the Link Worker was directly located inside the school. School-based LWs represent an important innovation in the general definition of SP whose focus is mostly on the primary care system.

*“The teachers, it’s good for them, because it’s quite an easy referral process. Whereas what was happening last year, as the teachers were just telling all our students to go to the doctors. So the teachers weren’t dealing with the problem in school, as soon as they realised there was a member of staff from door 43 accessible through the surgery. If a student came to them in crisis, they just told them to go to the doctor, which was frustrating because I think perhaps the school could have done more themselves first.”*

*LW 3*

### The networking and connecting role played by LWs

By its definition the encounter between Link Workers and their young clients is a co-productive relationship. Effective co-production with the young person relies on a shared understanding of Social Prescribing alongside a clear expectation of what Link Workers and clients can do together, including an in-depth conversation about the young person’s needs and challenges. The ultimate goal is to support young people in recognising what makes them feel well, how to find those resources in the community or in themselves and build the confidence to engage with local assets and, eventually, become independent.

*“My role focuses on working with you to understand how you feel and what matters to you. And create a personalised space based on these to help you to take control of your health and wellbeing. Things like loneliness, isolation, anxiety and low mood, difficulties in education, employment. And help you to connect with community groups, activities, support services, payments, to help you feel confident and continue independently.”*

*LW2*

The first Link Workers hired by Sheffield Futures created an efficient system that connects young people‘s wellbeing needs and values to community assets. The young person was invited to explore a list of different supporting social activities divided into six categories: creative, social, indoor, self-care, sports, vocational and educational. For each activity listed, Link Workers ensured the existence of an actual activity in the community through an ongoing mapping process and networking activity.

*“When I first came into the link worker role, I set it up so that for each activity that someone might take off, I had a service for each one. So if someone took that one off, I knew, right, there’s this service. And that was from researching what’s available, communicating with different services, to build the relationships to let them know, you know, I’m here, this is what I’m doing.”*

*LW1*

Beside working with the young person, Link Workers must engage and create a network they can refer to when linking the young person to the community. Networking comes through research but also through relationship building with the community assets.

*“I was searching what’s available, communicating with different services, to build the relationships to let them know that I’m here, this is what I’m doing. These are the kinds of people that I’m working with that I was asking to keep me updated with their services and any developments. So I can inform my young people as well. So we’ve got like resources where we’ve got lists of different services.”*

*LW2*

The networking activity is fundamental to allow Social Prescribing to be visible and recognized but also to share resources and information. In this sense the relationship that will be built between the young person and the community is facilitated by Link Workers.

*“It comes from just communicating with different kind of services in the city, to find out what there is really, and build the relationships, because when we support someone to go to a service, we don’t just want to say, here’s the information off you go, we want to kind of build that relationship between that young person and that service.”*

*LW1*

A strong feedback loop is developed between the Link Worker and the community assets and between the Link Worker and the young person which allows an ongoing evaluation of the connector scheme and what is available in the city. The relationship building between the community and link workers is seen as crucial in positive outcomes for the young person.

*“For me, it was going along to meetings with different services first to show who I am, what I do, get to know their service, and they can get to know our service. And then once I’ve got that relationship, if I then come across a young person who might benefit from their service, I can get in touch with that contact and say, I’ve got a young person, I think he will be interested, he’s facing these challenges, how can we come along?”*

*LW2*

Link Workers also report cases when the community is not flexible enough or it is not interested in co-producing with the Link Worker and the young person. This is seen as a limit in the community that would bring the young person to have a negative experience.

*“One of the organisation’s was the complete opposite. They were quite expensive, and they just prepared something without really checking what the needs were. And interestingly, the girls didn’t attend, only one girl went along to that and the others didn’t want to go on and that was the mistake they didn’t they weren’t really considered and consulted.”*

*LW3*

### Challenges faced by link workers in the delivery of the intervention

Link workers reported facing several challenges. The first challenge was what Link Workers call “inappropriate referrals” meaning the risk of being seen as a ‘gap filler’ for very vulnerable young people with high levels of mental health needs that have difficulties accessing support from statutory services such as CAMHS (Children and Adolescent Mental Health Services) which have very long waiting lists (from six to eighteen months).

*“So then the GP might think, “Well, this young person won’t be accepted into their service. So I’ll refer to the social prescriber”. But we then don’t have other services available that meet their needs.”*

*LW3*

The second key challenge link workers faced was maintaining the original meaning of Social Prescribing by providing social alternatives that can ultimately alleviate GPs caseload and mental health support for young people. They perceived a discrepancy between doctor’s expectations and young people’s needs in terms of mental health and the actual role of Social Prescribing.

*“Both misunderstanding and misuse. I just don’t think they realise the remit of the role. And then other times, I think they do realise, but they’re just hopeful that we would be able to fill the gap where there are other services, because they’re aware that the next services up from our threshold are really difficult to access, massive, long waiting lists and underfunded. So sometimes they just appear to be hopeful that we can do something, even if it’s not the perfect fit.”*

*LW3*

This discrepancy leads Link Workers to experience considerable frustration. In some cases, link workers can find solutions to young people’s mental health needs, when the burden is not too high and through the connection with trusted organisations in the community. In other cases, Social Prescribing is effectively delivered when the mental health need is supported by statutory services or by community assets.

An ongoing discussion in Sheffield Futures is about the need to better trained Link Workers able to provide basic level of support for mental health.

*“We have this ongoing debate about should we change our job description because what happens at door 43 is a lot of people are recruited to the roles and when they realise that it’s not strictly social prescribing, they leave the job. So time and time again, people* work *there for six months, and then they go. I feel like it’s because it’s falsely advertised because you think you’re going to do social prescribing, but then you end up being a mental health worker.”*

*LW1*

Link Workers felt that connecting young people with community-assets was not as easy as they initially thought. Most young people did not feel prepared or comfortable in engaging with local activities. During the pandemic, Covid-19, schools and activities closed forcing young people to spend long periods of time in their homes which became their comfort zone and discouraged them from re-connecting with people post-pandemic. Other young people were overwhelmed by extracurricular activities which prevented them to work on their self-esteem, anxiety and express their needs.

These challenges require Link Workers‘ autonomy and flexibility in dealing with each young individual according to their needs and aspirations. Link Workers follow young people from 6-12 sessions depending on the needs they can be more flexible. The length of the intervention depends on the level of self-esteem that the young person needs to acquire. Acquiring tools to manage their anxiety is an integral part of the Link Worker’s role.

Link Workers might signpost to social activities and activities more focused on healthier lifestyle if it is in the young person’s interest. They might also connect with local organisations that provide wellbeing services, counselling, LGBTQ+ community support, girls empowerment, and so on. In other cases, Link Workers might just suggest online tools for anxiety management, mindfulness and behavioural change.

*“People aren’t really ready to get involved in the community. We then offer kind of a, what we call like a phased approach where we might go for the first session. So say someone was interested in you know, arts and crafts like a group. We take them to the group, we might be there for the whole time. And then the second time, we might just meet them at the start at the venue. And then we might leave and they go in by themselves and they engage. So then they feel confident in, you know, accessing that support on their own.”*

*LW4*

## 5. Discussion

This paper described the role of Sheffield Futures Link Workers, from their profiles, the pathways into the intervention, how they identify and address young people’s needs and the connection with the community.

There is a current gap in the literature regarding the profiles of Link Workers working with young people. The paper has extracted common characteristics that are useful for defining this role. Link Workers participating in the research decided to apply for this job as they felt that their previous roles were too prescriptive, medicalized and assessment based, whilst in Social Prescribing the focus of their work is on the interests and needs of young people.

We described the three referral models that allow the intervention to be reachable and accessible, covering the whole city and to have a broader range of identifiers. This is an important contribution to the current literature as detailed descriptions of different referral models are particularly rare, even in the wider social prescribing literature. School-based Social Prescribing is a good example of how Social Prescribing for young people requires extending the definition to other identifiers and locations, besides the primary care setting.

The research shows how the Sheffield Futures Social Prescribing service is attempting to address the significant mental health challenges of young people by working with multiple actors and locating Link Workers in multiple spaces that are relevant for young people. The development of multiple referral pathways is an important innovation in social prescribing that is likely to benefit young people and the effectiveness of the system. The risk assessment tool should help identifiers, referees and Link Workers to assess who can benefit from Social Prescribing and avoid the intervention to become a gap filler for the lack of access for mental health support. As Social Prescribing for young people is a novel intervention, Link Workers are still working on the buy-in and understanding of what Social Prescribing is with the identifiers.

Link workers at Sheffield Futures recognize some discrepancy in the definition of their roles, as in some circumstances they might have to work as substitutes for mainstream mental health services (e.g. CAMHS) for which Social Prescribing might not be the adequate path. Similar to adult Link Workers [17, 18], this raises an important issue related to the responsibilities that Link Workers can have and consequently the role that community and healthcare practitioners should have when dealing with a mix of clinical and social needs.

The work of Link Workers in connecting young people to activities is not as obvious as it is in Social Prescribing for adults [19]. Young people might be overwhelmed by activities or might not feel prepared to get connected. In the co-production of the social prescription, Link Workers provide a deeper support to young people in building their self-esteem and sometimes there is no connection with social activities rather a networking with other mental health and wellbeing services. This is mostly true in those young people who are already overwhelmed by activities and do not need more, rather what they need is more support for their wellbeing. Link Workers highlight that in some cases there is no service in between CAMHS and other low-level mental health services as the same services provided by Sheffield Futures (I.e., counselling, wellbeing sessions). It also tends to happen that there is no connection at all with services in the community and that the young person is more in need to receive a listening space and strategies to deal with their emotional distress.

The programme developed by Sheffield Futures is continuously re-adapting and changing, to rapidly respond to the issues and needs that arise from the surrounding social context, the overload of healthcare systems and simultaneously ensure that the fidelity to the original meaning of Social Prescribing is maintained. With their practical work, Link Workers are operating at the forefront, capable of analysing the workarounds and barriers that were not immediately visible when setting up the intervention.

Sheffield is a vibrant city, with a good offer of services and activities for young people. The problem of onward referral is not per sé a barrier. Differently from adult Social Prescribing, in this case the main challenge is not the lack of capacity in the community, rather a community that lacks flexibility or is not sufficiently ready to co-design tailored offerings and activities. Community engagement in people’s wellbeing through Social Prescribing is an important feature and outcome. At Sheffield Futures the potential role that Link Worker plays by facilitating young people’s participation in community development is visible.

### Lesson Learned

Social Prescribing for young people delivered by Sheffield Futures has shown interesting aspects related to the role of link workers:

1. Young people referred to the service show the need for emotional support related to anxiety and low mood influenced by pressure from school performances, peers and families.
2. It is crucial to identify multiple referral pathways and focus on how multiple actors can be identifiers beside the primary care system can adapt Social Prescribing. School-based Social Prescribing is an experiment at Sheffield Futures, where the Link Worker role funded by a local PCN is located at school in order to intervene earlier.
3. The overall healthcare system is overloaded and its siloed structure does not allow immediate response to young people with low levels of emotional distress and mental health needs. There is the risk that without appropriate policy changes Social Prescribing might be adopted as a gap filler for broader systemic problems.
4. There is currently no direct interaction between social prescribing and CAMHS, which resonate with the non-clinical and not therapeutic work done by Link Workers. But there seems to be a connection between GPs’ demand for more mental health support, Link Workers case overload and waiting lists for CAMHS.
5. The intended aim of connecting people with community assets requires Link Workers to build trustful relationships with young people in order to strengthen their self-esteem and confidence prior to any possible connection with activities provided by the community. The majority of services to which young people are connected are related to mental health and emotional wellbeing. In some cases there is no connection at all with local resources which open the question regarding the suitability of Link Worker role.

**Figure 5:**
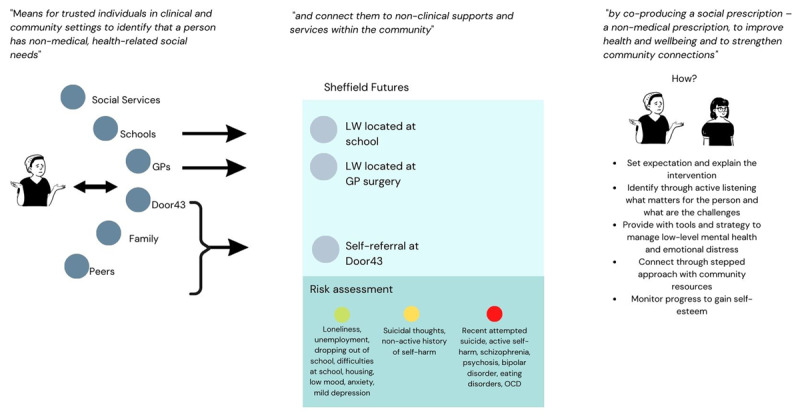
Visual synthesis of Link Workers’ role at Sheffield Futures.

We resumed through a visual synthesis the findings of the role of Link Workers based on the international definition of Social Prescribing (Figure 5).

### Further directions

Policy makers attention could be orientated toward the following assets:

1. Providing specialised mental health or emotional support training to support Link Workers in their work with young people would be particularly useful as their current skills might not be sufficient to deal with specific challenges young people face.
2. Develop an integrated care system which includes CAMHS, PCN, Social Prescribing and schools to better optimise support for young people in the waiting list for CAMHS. This could be done through a stepping-down model for young people who are already engaged in the mental health system or are on the waiting list. Although Social Prescribing does not assess mental health conditions, it can function as an integrated care intervention by focusing on the social circumstances which affect health and wellbeing in coordination with mental health services.
3. Support the development of school-based Social Prescribing through the involvement of PCN funding as a further connection across systems around the young person.

### Further research: the international use of Link Workers

Social Prescribing is gaining popularity globally and further research should focus on the understanding of the international use of this role and how it is developing outside the UK.

Recent research is exploring the role in different settings. For example in Australia [20] the link worker model has been recently adopted and evidence suggests the need to avoid inappropriate referral. This aspect highlights how a shared understanding of Social Prescribing across the community is needed to provide the correct support and avoid link workers to become gap fillers.

There is also a growing interest in the Social Prescribing Link Worker model for vulnerable young people worldwide, tackling a wider range of challenges not limiting the model as a mental health service. Further research should also consider the international use of Link Workers for young people.

## Conclusion

This paper focuses on the role of Link Workers as the core element of Social Prescribing but as it has already been suggested by the literature, it could also be interesting to investigate other models where the identifier might be a different actor (e.g., school staff) or a model without Link Workers. This will support the understanding of the mechanism that brings value in Social Prescribing. The National Health Service in England has recently introduced ‘proactive’ Social Prescribing which is an attempt to develop different models of Social Prescribing effectively by-passing the GP practice as main referrer of individuals to Social Prescribing support.
